# Current postoperative nutritional practice after pancreatoduodenectomy in the UK: national survey and snapshot audit

**DOI:** 10.1093/bjsopen/zrae021

**Published:** 2024-03-21

**Authors:** James M Halle-Smith, Samir Pathak, Adam Frampton, Sanjay Pandanaboyana, Robert P Sutcliffe, Brian R Davidson, Andrew M Smith, Keith J Roberts, REBOUND Study Group, Colin Wilson, Viswakumar Prabakaran, Asma Sultana, Ayesha Khan, Muhammad Butt, Declan Dunne, Melissa Bautista, Richard Laing, Dhanny Gomez, Raaj Praseedom, Michael Feretis, Giuseppe Kito Fusai, Gulbahar Syeda, Murali Somasundaram, Omar Mownah, Srikanth Reddy, Ali Arshad, Tayseer Al-Tawarah, James Skipworth, Jonathon Rees, Somaiah Aroori, Deborah Cipriani, James Milburn, Anya Adair, Maria Coats, Bilal Al-Sarireh, Oonagh Griffin, Nabeel Merali, Isabel Miglior, Rajiv Lahiri, Mary Phillips, Sarah Powell-Brett, Lewis Hall, Rupaly Pande

**Affiliations:** Hepatobiliary and Pancreatic Surgery Unit, Queen Elizabeth Hospital Birmingham, Birmingham, UK; College of Medical and Dental Sciences, University of Birmingham, Birmingham, UK; Hepatobiliary and Pancreatic Surgery Unit, Leeds Teaching Hospitals NHS Foundation Trust, Leeds, UK; Department of Hepato-Pancreato-Biliary Surgery, Royal Surrey County Hospital, Guildford, UK; Hepatobiliary and Pancreatic Surgery Unit, Newcastle Upon Tyne Teaching Hospitals NHS Foundation Trust, Newcastle Upon Tyne, UK; Hepatobiliary and Pancreatic Surgery Unit, Queen Elizabeth Hospital Birmingham, Birmingham, UK; College of Medical and Dental Sciences, University of Birmingham, Birmingham, UK; Hepatobiliary and Pancreatic Surgery Unit, Royal Free London NHS Foundation Trust, London, UK; Department of Surgical Innovation, Organ Regeneration and Transplant, University College London, London, UK; Hepatobiliary and Pancreatic Surgery Unit, Leeds Teaching Hospitals NHS Foundation Trust, Leeds, UK; Hepatobiliary and Pancreatic Surgery Unit, Queen Elizabeth Hospital Birmingham, Birmingham, UK; College of Medical and Dental Sciences, University of Birmingham, Birmingham, UK

##  

Despite advances in operative techniques and enhanced recovery programmes, morbidity after pancreatoduodenectomy (PD) remains high, with rates reported between 20% and 30%^1,2^. Perioperative malnutrition plays a key role in this, being associated with reduced quality of life and survival after surgery^3–5^. This is especially pertinent for PD patients, for whom malnutrition is present in a fifth before surgery^4^ and increases during inpatient stay to greater than 75% following surgery^3^. Nutritional management of PD patients is extremely complex, in part due to cancer cachexia caused by an aggressive malignancy but also due to the relative frequency of postoperative complications, such as delayed gastric emptying (DGE) and postoperative pancreatic fistula (POPF), which can limit oral intake. Strategies to improve the perioperative nutritional status of PD patients are urgently required. Designing a clinical trial requires a robust understanding of current clinician practice.

The aims of this study were to collect current clinician opinion and practice regarding nutritional management of patients after PD in the UK. An electronic invitation to participate in the clinician survey and snapshot audit was sent simultaneously to all pancreatic surgery centres in the UK. Institutional audit approval was obtained at each participating centre.

Of the 26 centres contacted, 19 responded to the electronic survey (73%) and in some cases, more than one clinician from each centre responded (23 clinician responses in total). Less than half of the centres had a written institutional standard protocol for feeding patients after PD (*n* = 8; 42%). Just under half of clinicians felt that early oral feeding was entirely safe (*n* = 11; 48%). In terms of oral feeding in patients with POPF, most clinicians felt it would be safe for patients with biochemical POPF (*n* = 17; 74%) but only 38% (*n* = 9) felt it would be safe for patients with clinically relevant POPF (CR-POPF), with the majority favouring nutrition with parenteral nutrition (*n* = 14; 61%). Most clinicians placed a nasogastric tube intraoperatively (*n* = 20; 87%), whereas just under half routinely used nasojejunal tubes (*n* = 10; 46%). Regarding immunonutrition, probiotics or synbiotics use, more than half of respondents were not aware of literature and felt more evidence was required (*n* = 14; 61%). Clinicians were broadly supportive of a potential RCT in which PD patients could be assigned to different perioperative nutritional interventions.

For the national snapshot audit, data from 12 centres were collected with 90 patients suitable for inclusion (*Table 1*). Most PD patients (*n* = 88; 98%) were allowed some form of oral intake on postoperative day 1 (POD1), with the most common modality being sips (*n* = 37; 45%). In terms of supplementary nutritional routes on POD1, 27% and 2% of patients received nutrition via nasojejunal tube and parenteral nutrition, respectively (*n* = 22 and *n* = 2, respectively). For those with biochemical POPF, 88% (15 of 17) continued to be fed orally with no change to their nutrition, whereas 76% (19 of 25) of those with CR-POPF were managed with parenteral nutrition and clear fluids orally. In terms of perioperative nutritional supplements, 15% of PD patients received immunonutrition whereas only 1% received probiotics (*n* = 14 and *n* = 1, respectively).

**Table 1:** Postoperative feeding practice reported in national snapshot audit

Nominal variables	Total (*n* = 90)
**Oral diet permitted on POD1**	
Sips	37 (45)
Clear fluids	26 (31)
Free fluids	16 (19)
Nil by mouth	2 (2)
**Alternative nutrition route POD1**	
Nasojejunal feeding	22 (27)
Parenteral nutrition	2 (2)
None	59 (71)
**NGT placed intraoperatively**	
Yes	83 (98)
No	1 (2)
**NGT removal day; *n* = 59**	
POD1	8 (14)
POD2	0 (0)
POD3	18 (31)
POD4	7 (12)
POD5	6 (10)
POD6	6 (10)
POD7−10	5 (9)
POD11+	9 (15)
**Biochemical POPF nutritional management; *n* = 17**	
Start nasojejunal feed and step down diet to free fluids orally	2 (12)
No change—continue oral diet	15 (88)
**CR-POPF nutritional management; *n* = 25**	
PN and step down diet to clear fluids orally	19 (76)
No change—continue oral diet	6 (24)
**Nutritional supplementation; *n* = 15**	
Immunonutrition	14 (15)
Probiotics	1 (1)
Synbiotics	0 (0)

Values are *n* (%). CR-POPF, clinically relevant postoperative pancreatic fistula; NGT, nasogastric tube; PN, parenteral nutrition; POD, postoperative day; POPF, postoperative pancreatic fistula.

This study reported the results of a national survey and snapshot audit of current perioperative nutritional practice after PD at UK pancreatic centres. The main findings were that there is widespread variation in perioperative nutritional practice and in opinion regarding nutritional management among this challenging patient group. The pancreatic surgery community in the UK seemed willing to investigate nutritional interventions in suitable clinical trials to generate further evidence in this area. Further understanding of whether the implementation of different interventions to address malnutrition is feasible among PD patients is required before a larger clinical trial can begin.

## Collaborators

Colin Wilson and Viswakumar Prabakaran (Freeman Hospital, Newcastle, UK); Asma Sultana, Ayesha Khan and Muhammad Butt (East Lancashire Hospitals, UK); Declan Dunne (Liverpool University Hospitals, UK); Melissa Bautista (Leeds University Hospitals, UK); Richard Laing (University Hospitals of North Midlands, UK); Dhanny Gomez (Nottingham University Hospitals, UK); Raaj Praseedom (Addenbrookes Hospital Cambridge, UK); Michael Feretis (Queen Elizabeth Hospital Birmingham, UK); Giuseppe Kito Fusai, Gulbahar Syeda and Murali Somasundaram (Royal Free Hospital, London UK); Omar Mownah (Kings Hospital, London, UK); Srikanth Reddy (Churchill Hospital, Oxford, UK); Ali Arshad and Tayseer Al-Tawarah (Southampton University Hospital, UK); James Skipworth and Jonathon Rees (University Hospital Bristol, UK); Somaiah Aroori and Deborah Cipriani (University Hospital Plymouth, UK); James Milburn (Aberdeen Royal Infirmary, UK); Anya Adair (Edinburgh Royal Infirmary, UK); Maria Coats (Glasgow Royal Infirmary, UK); Bilal Al-Sarireh (Morriston Hospital, Swansea, UK); Oonagh Griffin (St Vincents Hospital, Dublin, Ireland); Nabeel Merali, Isabel Miglior, Rajiv Lahiri and Mary Phillips (Royal Surrey Hospital, UK); Sarah Powell-Brett, Lewis Hall and Rupaly Pande (University Hospitals Birmingham, UK).

## Supplementary Material

zrae021_Supplementary_Data

## Data Availability

Anonymized data can be made available upon reasonable request.
