# Supplementary material for: Current postoperative nutritional practice after pancreatoduodenectomy in the UK: national survey and snapshot audit
Source: BJS Open. 2024 Mar 21;8(2):zrae021. doi: 10.1093/bjsopen/zrae021 (PMC10957164; doi:10.1093/bjsopen/zrae021)
Supplement: zrae021_Supplementary_Data [file zrae021_supplementary_data.docx]

**Title: Current Postoperative Nutritional Practice after Pancreatoduodenectomy in the United Kingdom – A National Survey and Snapshot Audit**

**Authors:** REBOUND Study Group

**Writing Group:** James M Halle-Smith^1,2^, Samir Pathak^3^, Adam Frampton^4^, Sanjay Pandanaboyana^5^, Robert Sutcliffe^1,2^, Brian Davidson^6,7^, Andrew M Smith^3^, Keith J Roberts^1,2^

**Institutions:**

^1^ Hepatobiliary and Pancreatic Surgery Unit, Queen Elizabeth Hospital Birmingham, Birmingham, United Kingdom

^2^ College of Medical and Dental Sciences, University of Birmingham, Birmingham, United Kingdom

^3^ Hepatobiliary and Pancreatic Surgery Unit, Leeds Teaching Hospitals NHS Foundation Trust, Leeds, United Kingdom

^4^ Department of Hepato-Pancreato-Biliary Surgery, Royal Surrey County Hospital,

Egerton Road, Guildford, United Kingdom

^5^ Hepatobiliary and Pancreatic Surgery Unit, Newcastle Upon Tyne Teaching Hospitals NHS Foundation Trust, Newcastle Upon Tyne, United Kingdom

^6^Hepatobiliary and Pancreatic Surgery Unit, Royal Free London NHS Foundation Trust, London, United Kingdom

^7^ Department of Surgical Innovation, Organ Regeneration and Transplant, University College London, London, United Kingdom

**Corresponding Author:**

James Halle-Smith

Email: james.hallesmith@doctors.org.uk

Postal Address: Hepatobiliary and Pancreatic Surgery Unit, 3rd Floor Nuffield House, Queen Elizabeth Hospital, Edgbaston, Birmingham, B15 2TH

Telephone: 01213714656

Facsimile: 01214141833

**Supplementary Materials - Index**

| **Supplementary Figures and Tables** |  |
| --- | --- |
| Supplementary Figure Legends | *pag. 2* |
| Supplementary Tables | *pag. 3-4* |
|  |  |

**Supplementary Figures and Tables**

**Supplementary Material**

***Supplementary Figure 1 – Clinician opinion and current practice for feeding on postoperative day 1 and feeding in postoperative pancreatic fistula after pancreatoduodenectomy***

*a) What is your opinion regarding early oral feeding (oral liquids on POD1, soft diet POD2) after PD?*

*b) What is the most common method of feeding on POD1 after PD at your institution?*

*c) What is your opinion regarding oral feeding in patients with biochemical and CR-POPF (assuming there is no evidence of DGE)?*

*d) What is your current practice regarding feeding for patients with biochemical and CR-POPF (assuming there is no evidence of DGE)?*

***
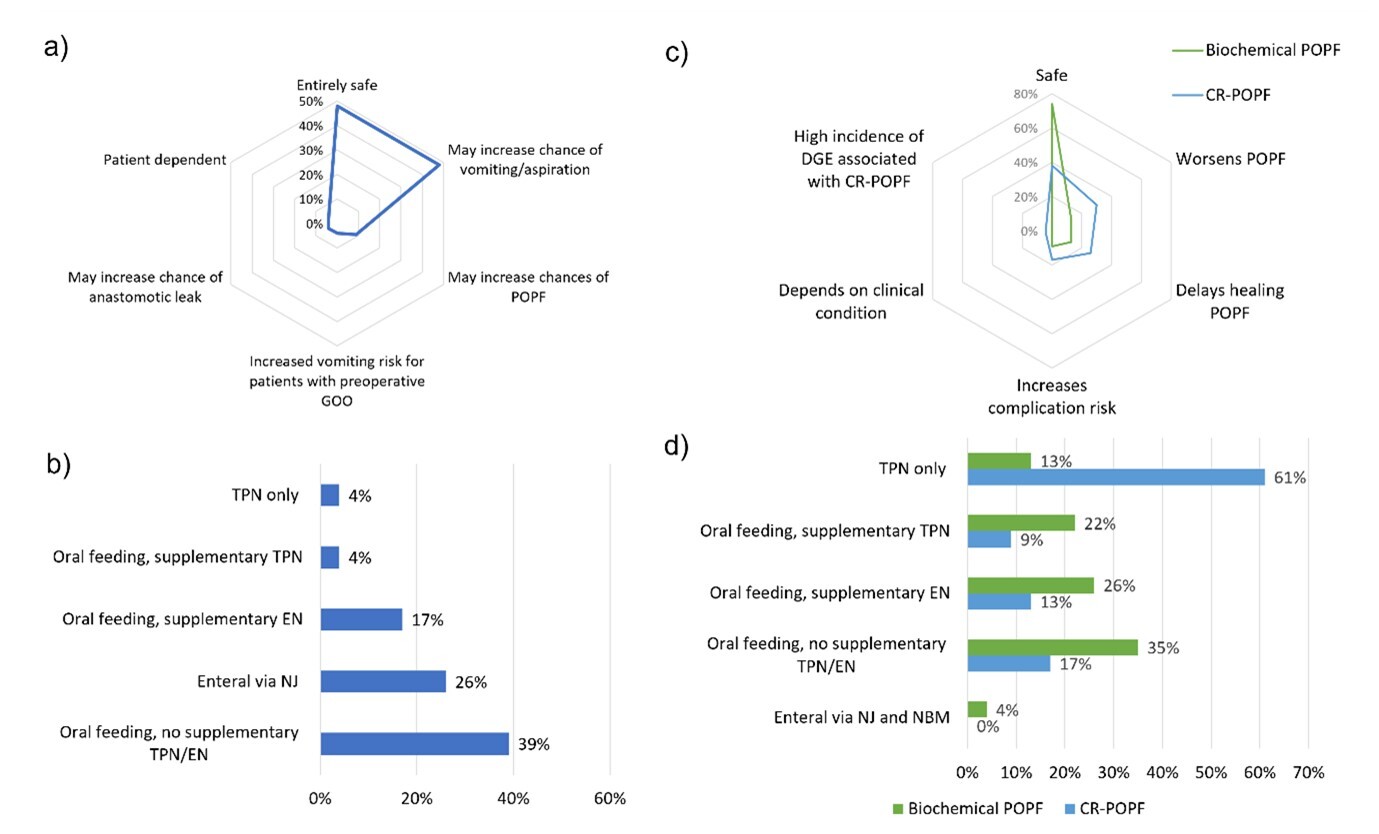
***

***Supplementary Figure 2 – Clinician opinion and Current Practice Regarding Nasogastric Tubes after Pancreatoduodenectomy***

*a) What is your opinion regarding use of NG tubes during pancreatic surgery?*

*b) If you place NG tubes routinely in PD patients, what is the main factor which determines when you will remove the NG tube postoperatively?*

***
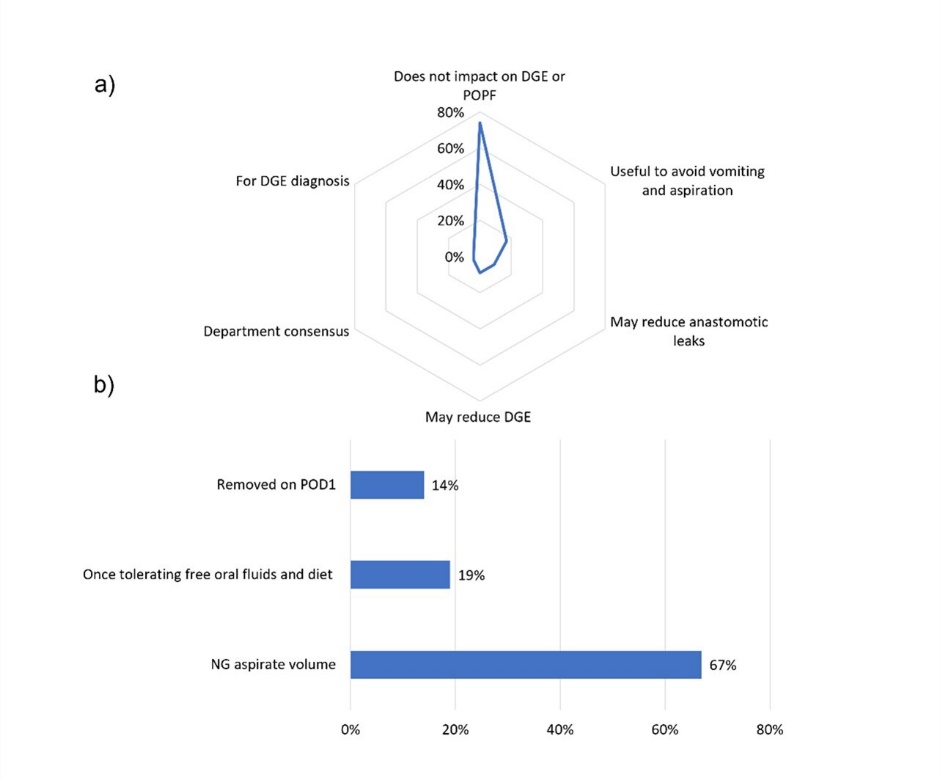
***

***Supplementary Figure 3 – Clinician Opinion and Current Practice for Nutritional Supplements after Pancreatoduodenectomy***

*a) What is your opinion regarding immunonutrition, probiotics and synbiotics in PD patients?*

*b) Do you use immunonutrition, probiotics or synbiotics routinely in your practice?*

***
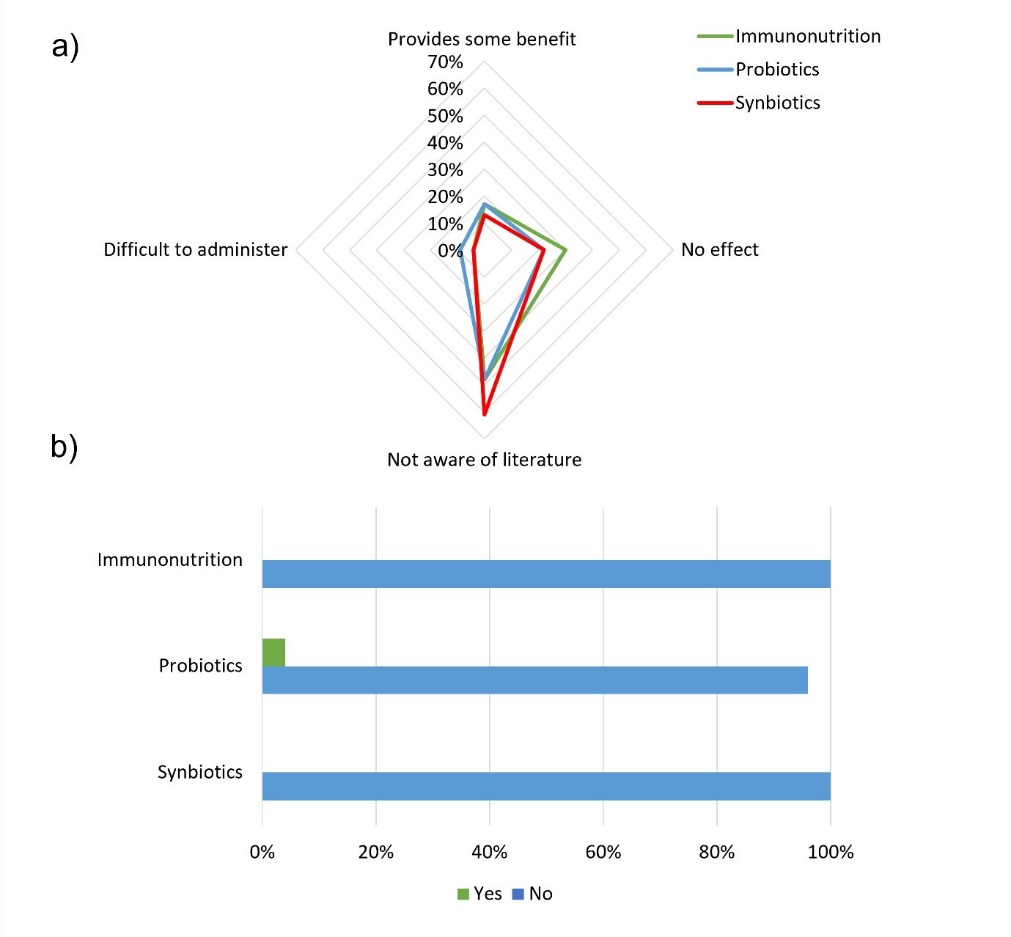
***

***Supplementary Figure 4 -* Acceptability of Perioperative Nutritional Interventions *after Pancreatoduodenectomy* in a Trial Setting**

*Would you be prepared to enter PD patients into a study where the following interventions were used in a treatment arm?*

***
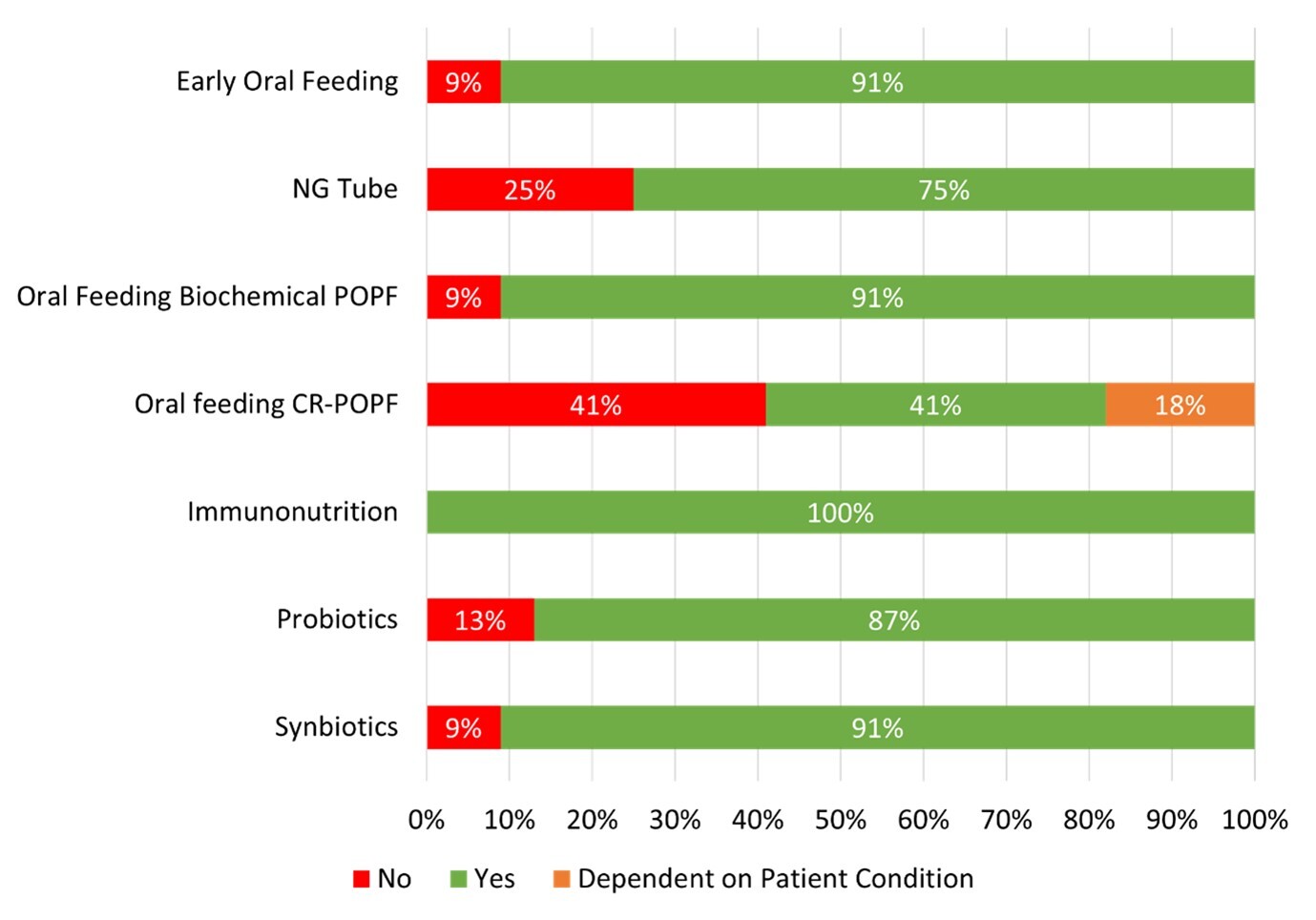
***

***Supplementary Table 1 – Pancreatic Centre Characteristics and Routine Practice***

*Nominal variables displayed as n (%)*

|  | **Number of Centres** |
| --- | --- |
| **Approximately how many PDs are performed at your institution per year?** |  |
| 0-25 | 1 (5) |
| 26-50 | 2 (11) |
| 51-100 | 9 (47) |
| 100+ | 7 (37) |
| **Does your unit follow an Enhanced Recovery after Surgery (ERAS) pathway which includes feeding and/or nasogastric tube management?** |  |
| No | 4 (21) |
| Yes | 15 (79) |
| **Do you have a published standard protocol for feeding patients after pancreatoduodenectomy?** |  |
| No | 11 (58) |
| Yes | 8 (42) |
|  |  |
|  | **Number of Clinicians** |
| **Which PD technique do you typically perform?** |  |
| Classical PD | 8 (35) |
| PPPD | 15 (65) |
| **Do you routinely administer octreotide (or other somatostatin analogue) after PD?** |  |
| Yes | 14 (61) |
| No | 9 (39) |
| **If you do administer octreotide routinely postoperatively, which dose do you use?** |  |
| 50 micrograms TDS | 1 (7) |
| 100 micrograms TDS | 12 (86) |
| 200 micrograms TDS | 1 (7) |
| **Do you routinely use NG tubes in patients undergoing pancreatoduodenectomy?** |  |
| Yes | 20 (87) |
| No | 3 (13) |
| **Do you routinely use NJ tubes in patients undergoing pancreatoduodenectomy?** |  |
| Yes | 10 (46) |
| No | 13 (54) |
| **Do you routinely use feeding jejunostomy tubes in patients undergoing pancreatoduodenectomy?** |  |
| Yes | 1 (4) |
| No | 22 (96) |

***Supplementary Table 2 – Demographics and Postoperative Variables in National Snapshot Audit***

*Nominal variables displayed as n (%) and continuous variables displayed as median (IQR)*

|  |  | **Total (n=90)** |
| --- | --- | --- |
| **Demographics** |  |  |
| Age |  | 66 (58-73) |
| Female | | 46 (53) |
| Charlson Comorbidity Score |  | 4 (3-5) |
| **Operative Characteristics** | |  |
| PD type | Classical | 40 (44) |
|  | PPPD | 50 (56) |
| Vascular resection | | 11 (12) |
| Indication | PDAC | 40 (44) |
|  | Duodenal | 8 (9) |
|  | Cholangiocarcinoma | 10 (11) |
|  | Ampullary | 14 (16) |
|  | Cyst | 3 (3) |
|  | NET | 6 (7) |
|  | Pancreatitis | 2 (2) |
|  | Other | 7 (8) |
| Postoperative level of care | 1 | 6 (7) |
|  | 2 | 63 (70) |
|  | 3 | 21 (23) |
| Somatostatin analogue postoperatively | | 77 (86) |
| **Postoperative Variables** | |  |
| Clavien-Dindo III+ | | 15 (17) |
| Delayed gastric emptying | Grade A | 14 (16) |
|  | Grade B | 10 (11) |
|  | Grade C | 6 (7) |
| Postoperative pancreatic fistula | |  |
|  | Biochemical | 17 (19) |
|  | Clinically relevant | 25 (28) |
| Length of Stay |  | 14.5 (9-23) |
| 90-day readmission | | 15 (17) |
